# Role of lung and gut microbiota on lung cancer pathogenesis

**DOI:** 10.1007/s00432-021-03644-0

**Published:** 2021-05-20

**Authors:** Yue Zhao, Yuxia Liu, Shuang Li, Zhaoyun Peng, Xiantao Liu, Jun Chen, Xin Zheng

**Affiliations:** 1 Shandong University of Traditional Chinese Medicine, Jinan, 250014 China; 2 Second Affiliated Hospital of Shandong University of Traditional Chinese Medicine, Jinan, 250001 China; 3 Qingdao Hospital of Traditional Chinese Medicine (Qingdao Hiser Hospital), Qingdao, 266000 China; 4 Affiliated Qingdao Central Hospital, Qingdao University, Qingdao, 266042 China; 5 Affiliated Hospital of Shandong University of Traditional Chinese Medicine, Jinan, 250014 China

**Keywords:** Lung cancer, Lung microbiota, Gut microbiota, Gut–lung axis

## Abstract

**Background:**

Lung cancer is the leading cause of cancer-related deaths worldwide (Ferlay et al., Int J Cancer 136:E359–386, 2015). In addition, lung cancer is associated with the highest mortality among all cancer types (Wu et al., Exp Ther Med 16:3004–3010, 2018). Previous studies report that microbiota play an important role in lung cancer. Notably, changes in lung and gut microbiota, are associated with progression of lung cancer. Several studies report that lung and gut microbiome promote lung cancer initiation and development by modulating metabolic pathways, inhibiting the function of immune cells, and producing pro-inflammatory factors. In addition, some factors such as microbiota dysbiosis, affect production of bacteriotoxins, genotoxicity and virulence effect, therefore, they play a key role in cancer progression. These findings imply that lung and gut microbiome are potential markers and targets for lung cancer. However, the role of microbiota in development and progression of lung cancer has not been fully explored.

**Purpose:**

The aim of this study was to systemically review recent research findings on relationship of lung and gut microbiota with lung cancer. In addition, we explored gut–lung axis and potential mechanisms of lung and gut microbiota in modulating lung cancer progression.

**Conclusion:**

Pulmonary and intestinal flora influence the occurrence, development, treatment and prognosis of lung cancer, and will provide novel strategies for prevention, diagnosis, and treatment of lung cancer.

## Introduction

Lung cancer is the leading cause of cancer-related deaths worldwide. The morphological, etiology and molecular characteristics of cancer have been widely explored (Aisner and Marshall 2012). In addition to genetic and environmental factors, microbiota play an important role in development of lung cancer. Microorganisms that occur in the human body are referred as “microbial communities”. Several microorganisms occur in human body and play a role in maintaining the dynamic and stable microenvironment in the host body. Although microbial communities are essential to human health, they can have detrimental effects on human health when the homeostasis is disturbed. Dysregulation of the microbiome is implicated in different diseases. Studies report that imbalance of microbial communities in specific organs is directly or indirectly associated with carcinogenesis (Garrett 2015; Khan et al. 2012). Microbiome dysbiosis affects susceptibility to carcinogenesis through multiple ways including modulation of the host inflammatory response, production of carcinogenic metabolic products, genotoxic and virulence effects and disruption of cell cycle (Mao et al. 2018). Microorganisms inhabit the gastro intestinal tract (GIT), lungs, skin, and other organs, with the gastro intestinal tract having the highest density of microorganisms. Microbial communities cause effects on the organs that they occur in and distant organs. Gut microbiome plays a crucial role in several diseases (Jobin 2012) especially in cancer. Moreover, gut microbiota may have important implications in the immune checkpoints treatment response and response to chemotherapy in NSCLC (Hakozaki et al. 2020). The role of lung microbiota in pulmonary disease has been widely explored through the recent advances in understanding of the respiratory immune system. Several recent studies have explored the relationship between lung microbiome and lung cancer (Dickson and Huffnagle 2015; Dickson et al. 2014b). In addition, the gut and lung form the gut–lung axis through complex bidirectional lymphatic and blood communication (Bingula et al. 2017). Therefore, we explored the correlation of lung and gut microbiota with development of lung cancer, and the effect of gut microbiome to therapy response of lung cancer. In addition, we explored the role of the gut–lung axis in carcinogenesis to provide new insights on the lung cancer pathogenesis. The study findings show that lung and gut microbes can be used as biomarkers for prevention, diagnosis and treatment of lung cancer.

## Lung microbiota and lung cancer

Traditionally, the lung was considered a sterile space. However, several studies report that unique microbial communities inhabit the lungs (Beck et al. 2012). Studies using 16S ribosomal RNA gene sequencing, both in physiological and pathological conditions showed that the lung hosts diverse microbiota. The microbiome of the lung mucous membrane is phylogenetically diverse (Erb-Downward et al. 2011; Hilty et al. 2010), compared with upper airways. A significantly lower number of bacteria inhabit lungs and lower respiratory systems compared with the gut (Segal and Blaser 2014). Lung microbiota has two dominating bacterial phyla in healthy adults including Bacteroidetes and Firmicutes (Morris et al. 2013; Segal et al. 2013). In addition, recent studies report that *Prevotella* and *Veillonella* inhabit healthy lungs (Hilty et al. 2010). Bacteria communities that inhabit lower respiratory system mainly include: *Megasphaera*, *Streptococcus*, *Pseudomonas*, *Fusobacterium* and *Sphingomonas* (Beck et al. 2012; Hilty et al. 2010). A recent study explored lung microbiota using surgical lung tissue samples reported that lung microbiota is unique, with *Proteobacteria* as the dominant phylum (Yu et al. 2016). Several microbial ecosystem studies using a wide range of biological samples such as sputum, lung tissues and bronchoalveolar lavage (BAL) fluid report that human lung comprises a diverse and low-density ecosystem of microbes. Several factors such as local conditions, migration of microorganisms down from the upper airways, and disposal of microorganisms by human affect microbiota composition (Dickson et al. 2014a). In healthy lungs, microbiota composition reflects microbial migration, reproduction, and elimination. Low concentrations of pulmonary microbiota are implicated in modulation of the immune system. An unbalanced ecosystem in the lungs may occur present as chronic respiratory diseases such as asthma and COPD, and may cause to pathogenesis of systemic diseases.

Previous studies report lung microbiome are implicated in development of lung diseases (Dickson and Huffnagle 2015; Dickson et al. 2014b). Several studies have explored the relationship between lung microbiota and lung cancer is gaining attention. However, most studies have not explored the mechanisms of lung microbiome on pathogenesis of lung cancer. Analysis of bronchoalveolar lavage fluid (BALF) from 20 patients with lung cancer showed that the relative abundance of two bacterial phyla (*Firmicutes* and TM7) and two genera (*Veillonella* and *Megasphaera*) was significantly higher in lung cancer patients compared with healthy individuals (Lee et al. 2016). A previous study using bronchoscopy samples from 210 lung cancer patients reported that high levels of Gram-negative bacilli *Escherichia coli*, *Enterobacter* and *Haemophilus influenzae* and Gram-positive cocci, *Staphylococcus* in lung cancer (Laroumagne et al. 2013). Yan et al. reported that salivary levels of microbiome *Capnocytophag*a and *Veillonella* were significantly high in lung cancer patients, implying that salivary microbiome is a potential biomarker for lung cancer (Yan et al. 2015). Female lung cancer patients in Xuanwei, China with no smoking history showed a higher level of *Granulicatella*, *Abiotrophia*, and *Streptococcus* genera in oral and sputum samples (Hosgood et al. 2014). A study involving lung cancer patients showed significantly higher abundance of *Streptococcus* in bronchoscopic specimens of lung cancer patients compared with healthy controls (Liu et al. 2018). Analysis of sputum samples of lung cancer patients showed significantly higher relative abundance of *Granulicatella adiacens* compared with the abundance in control subjects (Cameron et al. 2017).

In addition, studies report specific association between specific lung microbiota with different histopathological types of lung cancer. Therefore, these species can be used for diagnosis of different types of lung cancer. For instance, the saliva samples of 30 lung cancer patients was analyzed through high-throughput sequencing of 16S ribosomal RNA (rRNA) gene. The results showed that increasing titer of *Capnocytophaga*, *Selenomonas*, *Veillonella*, and *Neisseria* genera correlates with both adenocarcinoma (AC) and small-cell carcinoma (SCC), implying that these genera are potential biomarkers for lung cancer (Yan et al. 2015). Yu et al. reported that the microbiome composition of the squamous cell carcinoma tumor samples is different from and the microbial composition of adenocarcinoma (Dickson et al. 2014a). A recent study compared lung microbiota composition between patients with lung cancer and healthy controls and reported that unique lung microbiome is associated with tumor tissue. Squamous cell carcinoma cases with TP53 mutations showed higher abundance of *Acidovorax* taxa. In addition, *Klebsiella*, *Comamonas*, *Acidovorax*, *Polarmonas* and *Rhodoferax* genera are more frequently found in small-cell carcinoma (SCC), however, these associations are not seen in adenocarcinomas cases (Greathouse et al. 2018).

Several studies report involvement of the lung microbiota in pathogenesis of lung cancer (Laroumagne et al. 2013; Liu et al. 2018; Mao et al. 2018). One possible mechanism may be that bacteria cause chronic inflammation by promoting pro-inflammatory factors which stimulate airway epithelial cell proliferation, which ultimately induces cell transformation initiating tumor formation.

Moreover, some microbial components may confer a tumorigenic effect by directly affecting epithelial cells (oncogenes) (Ramirez-Labrada et al. 2020). Studies report that development of LC is related to local flora imbalance and inflammation. Pulmonary symbiotic flora causes inflammation associated with lung adenocarcinoma by activating γδ T cells that reside in the lungs. The incidence of lung adenocarcinoma was significantly reduced by elimination of the symbiotic bacteria. Symbiotic bacteria stimulate myD88-dependent IL-1B and IL-23 production in bone marrow cells, induce proliferation and activation of Vg6 + Vd1 + γδ T cells, mediate inflammation by inducing production of effector molecules such as IL-17, and lead to tumor cell proliferation in lung cancer (Jin et al. 2019a).

Studies should explore pulmonary microbial community as diagnostic and therapeutic markers of lung cancer. Although studies report association of lung microbiota with lung cancer, the mechanisms by which the pulmonary microbiome and the tumor interact have not been fully explored. Understanding the mechanism will provide information on the etiopathogenesis of lung cancer. Moreover, studies should design a method to accurately control systematic biases from sampling types and environmental contaminant. Different specimen types, such as lung tissues, sputum, BAL fluid and bronchoscopic samples may contain different microorganism types affecting results, therefore, it is important to establish unified standards using different sample collection and processing methods. Furthermore, sample sizes of some lung microbiome studies are too small, therefore, further large-scaled studies with longitudinal design and larger sample sizes should be carried out to identify and validate microbial biomarkers and explore microbiota-associated pathogenesis of lung cancer (Table. 1).

**Table 1:** Experimental evidence of relationship between lung microbiota and lung cancer

References	Variables	Results
Laroumagne et al. (2013)	Bronchoscopic samples	Gram-negative bacteria such as *Haemophilus influenzae*, *Enterobacter* spp. and *Escherichia coli*
Hosgood et al. (2014)	Oral and sputum samples from women in China	*Granulicatella*, *Abiotrophia*, and *Streptococcus* genera
Liu et al. (2018)	Bronchoscopic specimens	Genus *Streptococcus*
Cameron et al. (2017)	Sputum samples	*Granulicatella adiacens*
Lee et al. (2016)	Broncho alveolar lavage fluid	Two phyla (*Firmicutes* and TM7) and two genera (*Veillonella* and *Megasphaera*)
Yan et al. (2015)	Saliva samples	*Capnocytophaga*, *Selenomonas*, *Veillonella*, and *Neisseria* genera correlates with both SCC and AC
Greathouse et al. (2018)	Tumor tissue	*Acidovorax temporans* higher abundance of SCC

## Gut microbiota and lung cancer

Intestinal flora is the general term for all kinds of bacteria that inhabit the human gastrointestinal tract. Intestinal flora is widely distributed, complex and diverse microbial community. Intestinal flora maintain normal physiological and immune functions of the host intestinal tract, and catabolize food components making them easier to absorb. Gut microbiota has systemic effects on host physiology and health (Dickson et al. 2013; He et al. 2017; Samuelson et al. 2015; Tsay et al. 2011). Changes in intestinal flora composition (dysregulation), function, or interaction between the flora and the host are directly associated with several diseases. Several studies have explored the relationship between intestinal flora and various tumors. For instance, a previous study explored the role of gut microbiota in extra-gastrointestinal tumors (Fernández et al. 2018; Raza et al. 2019). In addition, studies report a relationship between lung cancer and gut microbiota (Gui et al. 2015; Routy et al. 2018; Zhang et al. 2018).

Zhuang et al. reported that elevated levels of Enterococcus in gut microbiota is associated with lung cancer (Zhuang et al. 2019). Moreover, an overall decline in gut microbial function occurs in lung cancer patients and *Enterococcus* and *Bifidobacterium* are reported to be potential biomarkers for lung cancer (Zhuang et al. 2019). Furthermore, lower levels of *Kluyvera*, *Escherichia-Shigella*, *Dialister*, *Faecalibacterium* and *Enterobacter* are reported in lung cancer patients, whereas *Veillonella*, *Fusobacterium* and *Bacteroides* are significantly higher compared with health individuals (Zhang et al. 2018). Gui et al. reported dysbiosis of gut butyrate-producing bacteria in non-small-cell lung cancer patients. In addition, gut butyrate-producing bacteria such as *Clostridium leptum*, *Faecalibacterium prausnitzii*, *Ruminococcus* and *Clostridial* cluster I spp showed significantly low levels whereas no change in abundance was observed for *Eubacterium rectal* and *Clostridial* Cluster XIVa in NSCLC patients (2020). Early-stage lung cancer is significantly associated with significantly lower relative abundance of gut microbiota including three phyla, 13 genera and 20 species whereas four phyla, 11 genera and 15 species are enriched. Furthermore, high levels of *Bacillus* and *Akkermansia muciniphila* promote development of lung cancer (Zheng et al. 2020). Liu et al. carried out a study involving 30 lung cancer patients, and reported low abundance of gut microbial community, and low biodiversity of microbial ecosystem, characterized by diverse and special pathogen microbiome and fewer probiotic genera (2019). Botticelli et al. reported that the level of *Prevotella*, *Lactobacillus*, *Rikenellaceae*, *Streptococcus*, *Enterobacteriaceae*, *Oscillospira* and *Bacteroides plebeius* in the stool of NSCLC patients was significantly higher compared with the level in healthy controls (2018).

Characteristics of gut microbiota in patients with lung cancer are different, implying that gut microbiota may affect lung cancer therapeutic and prognosis. A recent study explored the role gut microbiome to effectiveness of immunotherapy (Tartour and Zitvogel 2013). Implications of specific gut microbiome in cancer therapy have been explored through direct drug metabolism and modulation of the host immune response (Pouncey et al. 2018). Gut microbiota communities significantly affect immune checkpoint inhibitor therapy by regulating differentiation of regulatory T cells thus affecting immunomodulation mechanisms (Gopalakrishnan et al. 2018; Iida et al. 2013; Routy et al. 2018; Sivan et al. 2015; Viaud et al. 2013). Gut microbiome in lung cancer patients responding to treatment with immune checkpoint inhibitors differ significantly compared with those patients who showed no response to immunotherapy. Mice administered with fecal samples from responsive patients positively responded to immunotherapy, however, mice administered with fecal samples from non-responders did not respond to immunotherapy. Routy et al. reported significantly higher response to anti-PD1 therapy in lung cancer patients was positively correlated with abundance of *Akkermansia muciniphila* species. Another study reported that *Akkermansia* *muciniphila* is correlated with positive response to ICI treatment. Supplementation with *Akkermansia* *muciniphila* increased response to ICI whereas abnormal composition of gut microbiota is implicated in resistance to ICI treatment (Routy et al. 2018). Song et al. reported that diversity of gut microbiota is correlated with anti-PD-1 immunotherapy response (2020). Fecal samples comprising mainly *Proteobacteria*, *Firmicutes*, *Bacteroidetes* and *Actinobacteria* bacterial communities improved response to anti-PD-1 immunotherapy (Song et al. 2020). In addition, a previous study reported that the composition of gut microbiota is relatively stable and higher diversity is observed in NSCLC patients who responded to nivolumab (Jin et al. 2019b). Moreover, prolonged progression-free survival (PFS) is reported in patients with high microbiome diversity compared with patients with low diversity (Jin et al. 2019b). A study retrospectively evaluated 118 advanced NSCLC patients treated with immune checkpoint blockade, and reported that administration of *clostridium butyricum* therapy (CBT) before and/or after receiving immune checkpoint blockade therapy significantly prolonged PFS and overall survival (OS) in patients (Tomita et al. 2020).

In addition to improving response to immune checkpoint inhibitor therapy, gut microbiota affects chemotherapeutic efficacy in lung cancer. The effect of oral feeding of *Lactobacillus acidophilus* on cisplatin treatment was explored using lung cancer mouse models. Administration of *Lactobacillus acidophilus* enhanced antitumor effect of cisplatin, reduced the tumor size, improved survival rate. These findings imply that coadministration with probiotic improves the anti-growth and pro-apoptotic effects of cisplatin (Gui et al. 2015). Moreover, patients with late-stage *lung* cancers treated with *Enterococcus hirae* and *Barnesiella intestinihominis* in combination with chemo-immunotherapy showed longer progression-free survival (Daillère et al. 2016). Increase in survival in these patients can be attributed to improved immunomodulatory action.

In addition, studies report that high consumption of yogurt is associated with significant reduction in lung cancer risk (30%). These findings imply that prebiotics and probiotics have potential protective effects in lung carcinogenesis (Yang et al. 2020).

Change in diversity of gut is potential biomarker for diagnosis and treatment of lung cancer (Bai et al. 2019). However, the role of gut microbiome in development and progression of lung cancer should be explored further. In addition, role of microbiome in modulating the effectiveness of anti-cancer treatment should be analyzed further (Table. 2).

**Table 2:** Relationship between gut microbiota and lung cancer

References	Variables	Summary findings
Zhuang et al. (2019)	Fecal samples	*Enterococcus*
Zhang et al. (2018)	Fecal samples	Higher levels of *Bacteroides*, *Veillonella*, *Fusobacterium* but lower levels of *Escherichia-Shigella*, *Kluyvera*, *Faecalibacterium*, *Enterobacter*, *Dialister*
Gui et al. (2020)	Fecal samples	Gut butyrate-producing bacteria dysbiosis
Zheng et al. (2020)	Fecal samples	Elevated *Bacillus* and the *Akkermansia muciniphila*
Song et al. (2020)	Fecal samples	Phyla *Bacteroidetes*, *Firmicutes*, *Proteobacteria*, and *Actinobacteria*
Liu et al. (2019)	Fecal samples	*Enterobacteriaceae*, *Streptococcus*, *Prevotella*
Botticelli et al. (2018)	The stool of NSCLC patients	*Rikenellaceae*, *Prevotella*, *Streptococcus*, *Lactobacillus*, *Bacteroides plebeius*, *Oscillospira*, and *Enterobacteriaceae*

## Gut–lung axis and lung cancer

Although gastrointestinal and respiratory tracts are distant physically, they have the same embryonic origin and high similarity in structure*,* implying that the two sites might interact in multiple aspects. There is a clear cross-talk between gastrointestinal and respiratory tracts known as gut–lung axis, which was reported recently. Studies report that intestines and lungs interact with each other through microbial and immune functions to achieve two-way regulation. Gut and lung microbes show similar colonization characteristics in early days of life, and gut and lungs have strong mucosal defense system against microbes. Furthermore, intestinal mucosa and pulmonary mucosa show several similarities after differentiation. For example, intestinal mucosa goblet cells can secrete IgA, whereas goblet cells of respiratory mucosa can also produce IgA. In addition, studies report that lung and intestines can affect each other’s immunity (Gill et al. 2010). Previous studies report that short*-*chain fatty acids (SCFAs) which are the major metabolic products of gut microbiota from dietary fiber, have a mediating role of the gut microbiome’s immune function, in an allergy model, and can regulate lung immunity (Cait et al. 2018; Trompette et al. 2014). Moreover, bacterial lipopolysaccharide (Gray et al. 2017) and immune cells such as TREG cells regulate pulmonary immune response, thus affecting the microbes that colonize lungs (Samuelson et al. 2015). Gut microbiota can therefore, activate B and T cells and other immune cells, which infiltrate the lungs through hematogenous or lymphatic routes, and activate lung immune response. As a result, they induce numerous respiratory diseases, such as COPD, cystic fibrosis, respiratory infection and asthma (Bingula et al. 2017; Budden et al. 2017; Marsland et al. 2015; Shukla et al. 2017). Studies report that lung flora can affect intestinal flora through circulation of the blood (Renz et al. 2011). Gut and lung microbiota are linked to each other by a complex bidirectional axis through lymphatic (Bingula et al. 2017) and blood circulation system. For example, *Streptococcus pneumoniae* infected mice treated with antibiotics, showed that administration of healthy mice feces suspension can relieve symptoms of pneumonia. Oral administration of *lactobacillus* and *bifidobacterium* alleviates symptoms of childhood asthma and reduces seizure frequency. A study based on responses of Th17 cells, reported that viral infection in the lung induces intestinal injury (Wang et al. 2014). These findings show that changes in intestinal microorganisms affect immune responses in lungs and transformation of pulmonary diseases (Budden et al. 2017).

In summary, a bidirectional regulation of the intestinal-lung axis occurs between the intestine and lungs. Changes in this axis lead to deleterious outcomes, such as development of cancer, colonization of pathogens, damage of tissue and increased susceptibility to infections (Hooper et al. 2012; Mazmanian et al. 2005).

**Fig. 1:**
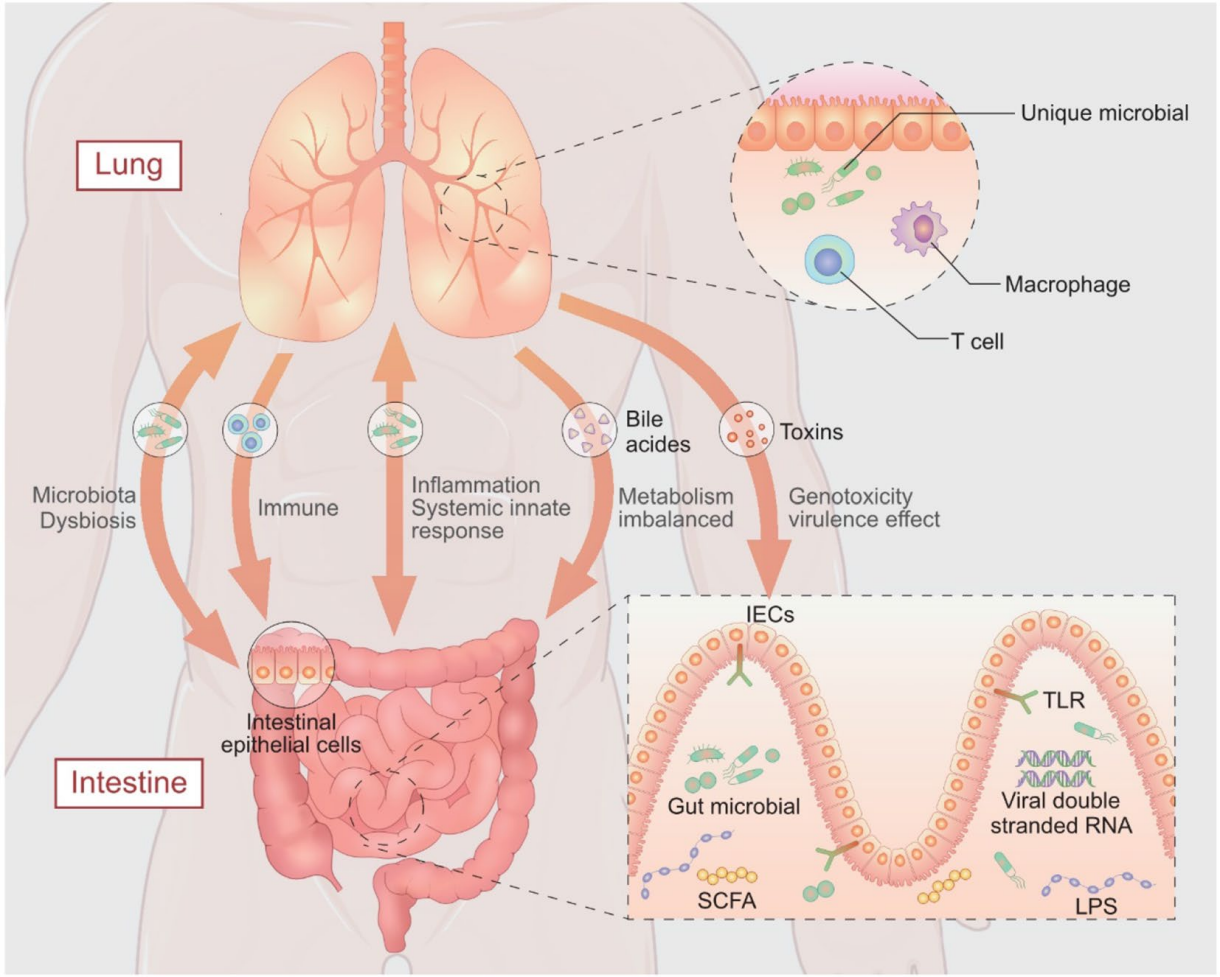
Role of gut–lung axis on lung cancer pathogenesis. Immune response: presence of immune cells in the submucosal layer or mesenteric lymph nodes. Different microbial ligands are recognized by Toll-like receptors (TLRs) on the surface of intestinal epithelial cells (IECs), thus activating TLR innate-adaptive immunity leading to differentiation and activation of T cells, and activation of macrophages and dendritic cells. Most migratory bacteria and their metabolites directly stimulate intestinal epithelial cells regulating immune cells. Migration of immune cells activates the mucous membranes in the gut. Inflammation: lung inflammation can trigger the systemic innate response, which increases susceptibility to inflammatory insult in intestines. Microbes or their products (such as SCFAs) migrate from the gastrointestinal tract across the mucosal barrier to bloodstream thus mediating inflammation. Altered metabolism: different microbial bioactive molecules released from microbiome can affect hosts metabolism. Deoxycholic acid and lithocholic acid are secondary metabolites produced from bile acids by intestinal bacteria which cause DNA damage and cancer initiation. Formation of toxic metabolites in the lungs due to imbalanced metabolism may contribute to lung carcinogenesis. Genotoxicity and virulence effect: imbalance and changes of bacteriome composition can produce a variety of toxins which promote free radical production. These radicals play a carcinogenic role in the host organism. Microbiota dysbiosis: imbalance in the microbiota mediates lung cancer initiation and development by enhancing production of inflammatory mediators and though production of cytotoxic substances

Microbiota can affect occurrence and development of cancer through multiple pathways. Currently, studies report that microbial community affect lung cancer by modulating immune response, inflammation, metabolism, genotoxicity and virulence effect. 1. Immune response: a large population of immune cells such as macrophages exist in the submucosal layer or mesenteric lymph nodes in the intestinal tract, and gut microbiota provide pathogen-associated molecular patterns (PAMPs) from different microbial origin (Samuelson et al. 2015). Toll-like receptors (TLRs) on the surface of intestinal epithelial cells (IECs) are pathogen-associated recognition receptors, which recognize different microbial ligands, such as lipopolysaccharide (LPS) or viral double-stranded RNA, or toxin from parasites and fungi. If microbial ligands are not eliminated by the first line of defense, the protein portion of the surviving or dead bacteria and fragments of cell wall can infiltrate the cisterna chyli through the mesenteric lymphatic system, escape cytokines and chemokines produced in the gut, and enter the intestinal circulatory system. These ligands enter into pulmonary circulation and activate TLR innate-adaptive immunity leading to differentiation and activation of T cells, and activation of macrophages and dendritic cells. Moreover, migratory bacteria have been reported in commensal bacteria and their metabolites, such as SCFAs, propionate and butyrate directly stimulate intestinal epithelial cells thus regulating release of immune cells. Furthermore, migration of immune cells activates mucous membranes in the gut, the first place where antigens meet. 2. Inflammation: lung inflammation affects intestinal microbiota and blood microbiome affects through the gut–lung axis (Dumas et al. 2018)*.* However, the role of lung inflammation on pathological responses within intestinal tissue should be explored far to understand the mechanism. Kim et al. used an animal model of short-term smoke exposure and reported that lung inflammation triggers systemic innate response, which increases susceptibility to inflammatory effects in the intestine (Kim et al. 2019). Bacterial translocation resulting in transfer of microbes or their products from the gastrointestinal tract across the mucosal barrier to the bloodstream is implicated in inflammation (Schuijt et al. 2016). A previous study reports that feeding mice with a high-fiber diet reduces inflammatory cell infiltration, thus enhancing protection against allergic pulmonary inflammation (Trompette et al. 2014). Gut microbes decompose dietary fiber with the aid of digestive enzyme thus promoting intestinal absorption of short-chain fatty acids (SCFAs). SCFAs are a group of metabolites implicated in inflammation. Treg suppresses airway inflammation by stimulating SCFAs. 3. Altered metabolism: different microbial bioactive molecules from microbiome can affect host metabolism. For instance, deoxycholic acid and lithocholic acid which are secondary bile acids produced from bile acids by intestinal bacteria, cause DNA damage and are implicated in cancer initiation (Louis et al. 2014). In addition, carcinogens such as acetaldehyde (Madan et al. 2012) and deoxycholic acid (Trompette et al. 2014) are implicated in pathogenesis of esophageal and liver cancer. Formation of toxic metabolites in the lungs due to imbalanced metabolism may contribute to lung carcinogenesis. 4. Genotoxicity and virulence effect: imbalance and changes in bacteriome composition, which are considered to be genotoxic, can produce a variety of toxins, promote free radical production, cause DNA lesions, cell cycle arrest and apoptosis without DNA repair. As a result, changes in microbiome play a carcinogenic role in host organism (Druzhinin et al. 2018). 5. Microbiota dysbiosis: a previous study reports that antibiotic treatment modifies the balance of commensal microbiota, alters the population and composition of the microbiota, and increase risk of developing lung cancer (Boursi et al. 2015). Therefore, imbalance of microbiota is highly correlated with occurrence of lungcancer. Moreover imbalance in microbiota and pathogenic bacterial flora termed dysbiosis are involved in lung cancer initiation and development by enhancing production of inflammatory mediators and production of cytotoxic substances (Fig. 1).

These findings show that a complex network exists in gut–lung axis, linking immune responses between the lungs and the gut. An hypothesis based on the gut*–*lung axis theory states that stimuli derived from gut, is implicated in protective effects in lungs (Bingula et al. 2017). In addition, gut microflora changes affect lung microbiota alterations, which in turn affects gut microbiota through the blood circulatory system (Budden et al. 2017).The gut–lung axis explains the complex bidirectional communication system between the gut and lungs. The stimuli in the gut affects the lung through the gut*–*lung axis mechanisms, whereas lung feedback and altered signals are released to the gut through the gut–lung axis.

However, the mechanism underlying the role of gut–lung axis in pathogenesis and progression of lung cancer and the potential for manipulation of the gut–lung axis in the treatment of lung cancer should be explored further. These studies show that lung bacterial represents a major component of the lung tumor microenvironment and the gut–lung axis allows indirect modification of lung bacterial composition through gut microbiota modification strategies, such as fecal transplantation. A previous study reports that TNF-α induces epithelial-to-mesenchymal transition thus promoting lung cancer metastasis (Shang et al. 2017). Bifidobacterium, a representative of intestinal probiotics has multiple physiological activities, which are protective against TNF*-*α and lipopolysaccharides (LPS) induced inflammatory thus conferring anti-cancer effects (Boesten et al. 2011). Gut or lung microbiota modification strategies, such as a direct translocation of bacteria from one site to the other, release of bacteria-derived immunomodulatory molecules into the blood stream and the lymphatic system thus affecting systemic immunity, can be used to achieve dynamic crosstalk between the two sites (Barfod et al. 2013; Milani et al. 2017; Schuijt et al. 2016; Vallès et al. 2014). Crosstalk between the microbiota of gut–lung axis provide potential new therapeutic targets for lung cancer. However, the underlying mechanism of the connection between the gut–lung axis and lung cancer should be explored further.

## Conclusions and future perspectives

The human body is a complex habitat for diverse microorganisms. The human flora helps to maintain the overall homeostasis of the host. Pulmonary and intestinal flora influence the occurrence, development, treatment and prognosis of lung cancer. Changes in lung and gut microbial functions and microbiome associated molecular patterns (MAMPS) caused by immune processes, inflammation, and bacterial dysbiosis *are* the driving mechanisms of lung cancer. Thus, the role of gut–lung axis in the pathogenesis of lung cancer get has attracted huge attention. Moreover, future randomized controlled trials and real world studies should be conducted with improved methodologies to determine the clinical value of the microbiota-cancer relationship and elucidate how the microbiome affects lung cancer. This will reveal promising diagnostic and therapeutic avenues. We hope that lung and gut microbes can be used as biomarkers for the assessment of the progression and guide the treatment of lung cancer, as well as provide alternative targets for cancer prevention. It also can be expected that “design probiotics” and other means to regulate the flora, so as to improve the curative effect and prognosis of lung cancer patients.
